# The impact of nutritional counseling in the healthy diet score in type 1 and type 2 diabetes

**DOI:** 10.1186/1758-5996-7-S1-A239

**Published:** 2015-11-11

**Authors:** Rodrigo Bastos Fóscolo, Ann Kristine Jansen, Carolina Jovita Oliveira Dias

**Affiliations:** 1 UFMG, Belo Horizonte, Brazil

## Background

In the last decade in Brazil, the inadequate nutrition appears as the main risk factor for disability related chronic diseases.

## Objective

To evaluate the impact of nutritional counseling in the healthy diet score in type 1 diabetes (T1D) and type 2 (DM2).

## Materials and methods

Intervention study with 39 adult diabetic patients of both sexes after a year of individual clinical and nutritional monitoring, the multidisciplinary outpatient Diabetes League, 19 DM1 and DM2 20. During the research the patients were instructed to change their eating pattern according to the recommendations of the American Diabetes Association. We collected data from medical records at the first visit and one year after. Clinical, biochemical, anthropometric and food consumption were evaluated. Food consumption was assessed by a healthy diet score that classifies food into three categories of variables: Ideal (score 2), intermediate (score 1) and poor (score 0). The maximum score possible was 16, considering ideal: Daily consumption of foods rich in monounsaturated fatty acids, at least 2 servings of whole grains and 400 grams of vegetables fruits and vegetables; three times a week fish, at most once a week processed foods high in sodium, high in animal fat, fried foods and soft drinks. The score reliability test was satisfactory (Cronbach's alpha=0.614). Statistical analysis was performed with SPSS 17.0 software.

## Results

At baseline the DM1 group's average score was 5.89 (±2.48) and DM2 5.98 (±2.93). At end, both groups presented significant increase (p <0.05), and the average DM1 was 9.66 (±1.80) and DM2 8.24 (±2.61). There was no significant difference when comparing the scores between the two groups (p> 0.05). It was noted also better diabetes control in both groups after one year follow up. The DM1 used far less time insulin dosage (p=0.042), and type 2 diabetes had greater levels of HLD-c (p=0.017) and reduced systolic blood pressure (p=0.048). There were no significant anthropometric changes.

## Conclusion

The nutritional and clinical follow-up, within one year, can increase the healthy diet score in diabetes, contributing to improved dietary pattern. In addition, it favored diabetes control and decrease in comorbid risk.

